# Acute Effects of Two Types of Dumbbell Exercise on Oxygenated Hemodynamic Concentration of Cerebral Activation in Healthy Young Male Adults: A Functional Near-Infrared Spectroscopy Study

**DOI:** 10.3389/fnhum.2020.519171

**Published:** 2020-11-05

**Authors:** Yana Wang, Jiaojiao Lü, Jifeng Rong, Linjie Song, Wei Wang, Yifan Jiang, Yu Liu, Lingyan Huang

**Affiliations:** ^1^Key Laboratory of Exercise and Health Science of Ministry of Education, Shanghai University of Sport, Shanghai, China; ^2^Department of Rehabilitation Medicine, Shanghai First Rehabilitation Hospital, Shanghai, China

**Keywords:** momentum dumbbell, conventional dumbbell, strengthening exercise, functional near infrared spectroscopy (fNIRS), surface electromyography (sEMG)

## Abstract

**Purpose**: To examine cerebral cortical activation differences in the frontal cortex and parietal lobe during the performance of two types of dumbbell exercise.

**Methods**: A total of 22 young healthy male adults (mean age, 23.8 ± 2.05 years; height, 1.75 ± 0.06 m; weight, 71.4 ± 8.80 kg) participated in a crossover design study that involved two experimental exercise conditions: momentum dumbbell and conventional dumbbell. Performance tasks included 10, 10-s sets of single-arm dumbbell exercise, with a rest interval of 60 s between sets and a 5-min washout period between conditions. The primary outcome was the cerebral concentrations of oxygenated hemoglobin (HbO_2_) in the frontal cortex and parietal lobe assessed during performance of both exercises using functional near-infrared spectroscopy (fNIRS). The secondary outcome was upper-limb muscle activation measured using surface electromyography (sEMG). Outcome data were ascertained during exercise.

**Results**: A significant between-condition difference in HbO_2_ was observed in the frontal and parietal regions with an increase in HbO_2_ during momentum, relative to conventional, dumbbell exercise (*p* < 0.05). Compared to conventional dumbbell exercise, performing a momentum dumbbell exercise led to a higher level of muscle activation in the anterior and posterior deltoids of the upper arm and in the flexor carpi radialis and extensor carpi radialis longus of the forearm (*p* < 0.05). However, no between-condition differences were found in the biceps and triceps brachii (*p* > 0.05).

**Conclusion**: Dynamic, compared with conventional, dumbbell exercise resulted in higher hemodynamic responses and greater upper-limb muscle activation in young healthy adults. The findings of this study showed differential cortical hemodynamic responses during performance of the two types of dumbbell exercise with a higher activation level produced during momentum-based dumbbell exercise.

## Introduction

There is now irrefutable scientific evidence showing the health benefits of various exercise modalities such as aerobic, resistance and balance training and flexibility exercise in improving cardiovascular fitness (Fletcher et al., 2018), cognitive function (Northey et al., 2018), musculoskeletal health (Hagen et al., 2012), and the metabolic system (Park and Larson, 2014). Among the most common exercise modalities, resistance training, which causes the muscles to contract against an external resistance, has been shown to be therapeutically effective in improving muscular strength (Grgic et al., 2018), balance (Orr et al., 2008), bone health (Hong and Kim, 2018), and cognitive function (Li et al., 2018) among younger and older adults. In addition, resistance training regimens such as free-weight dumbbells were also shown to elicit changes in the hemodynamic concentration of the primary motor cortex in healthy adults (Bai et al., 2016).

Using a novel momentum dumbbell exercise type that involved self-initiated spinning of a handheld dumbbell with a built-in pendulum to generate momentum for training upper-limb strength and core stability, Lü et al. (2015) evaluated the effects of this motor–cognitive integrated dynamic exercise on cognitive function among older adults with mild cognitive impairment. Findings of the study showed that, compared with a no-exercise control condition, 12 weeks of momentum dumbbell training resulted in significant improvement in cognitive ability and physical performance. Thus, while conventional dumbbell exercise was shown to increase brain oxygenation (Bai et al., 2016), the augmented momentum dumbbell exercise has also shown potential for enhancing cognition.

Therefore, by extending the studies of conventional (Bai et al., 2016) and momentum (Lü et al., 2015) dumbbell exercises on brain activity and cognitive function, this study aims to examine cerebral cortical activation differences in the regions of the frontal cortex and parietal lobe during the performance of conventional dumbbell and momentum dumbbell exercises using functional near infrared spectroscopy (fNIRS) among young healthy adults. We specifically targeted the frontal cortex and parietal lobe because of their functional (motor and sensory) roles intrinsically linked with exercise performance (Bai et al., 2016). We hypothesized that, compared with conventional dumbbell exercise, momentum dumbbell exercise would lead to a higher level of oxygenated hemoglobin (HbO_2_) concentration in the frontal cortex and parietal lobe. As secondary outcomes, we also examined the differences between these two exercises on upper-arm muscle activation patterns.

## Materials and Methods

### Study Design and Participants

The study used a crossover design in which 22 male adults completed two different dumbbell-based exercise conditions: momentum dumbbell exercise and conventional dumbbell exercise, consecutively administered in each participant. Eligibility criteria included being male, age ≥20 years, absence of neurological disorders, and physical fitness. Subjects were recruited through public promotion and word of mouth among college students studying at the Shanghai University of Sport (SUS) in Shanghai, China. All participants provided written consent to participate in the study, and the study procedures were approved by the Ethics Committee at SUS and conformed to the Declaration of Helsinki.

### Experimental Tasks

**Figure 1:**
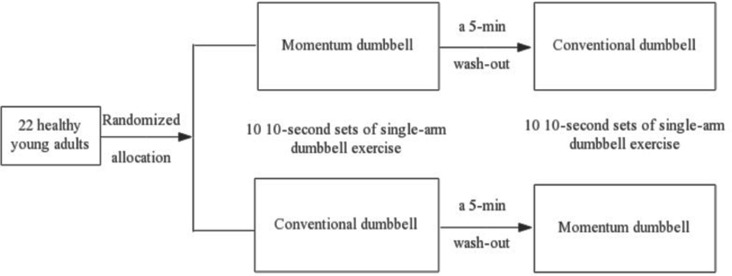
Flow of the cross-over study design.

Participants were instructed to stand straight with their feet hip-width apart, toes facing forward, and handhold a dumbbell (in a horizontal position) using the dominant hand with palms facing up, positioned on the lateral side of the body. Participants were asked to stand quietly for 60 s and, upon receiving a “Go” signal light (placed in front of them), to begin performing the single-arm dumbbell curl exercises at their self-selected speed for 10 s. Each dumbbell exercise session consisted of 10 10-s sets of exercise with a 60-s resting break in between. A 5-min interval (wash out) between the two dumbbell exercise conditions was provided. Figure 1 describes the flow of the current study.

The momentum dumbbell used in this study was a handheld device with two built-in eccentric pendulums (approximately 9 cm in length) at each end, with dimensions of 32 cm in length and 22 cm in diameter and weighing 1.92 kg (Lü et al., 2015). During the exercise, participants were instructed to perform a continuous spinning motion by having the pendulums inside the dumbbell spinning in a clockwise direction around the frontal axis for up to 10 s. For the conventional single dumbbell exercise, participants were instructed to engage in bicep curls performed in the sagittal plane at their own pace without moving the upper arm by bending the elbow joint and curling the dumbbell at approximately 90 angles, then allowing the dumbbell to return to the starting position. A brief cool-down period was provided at the end of the exercise experiment.

### Experimental Procedure

The experiment was conducted at a research laboratory located in an academic setting. Participants reported to the laboratory twice, at an interval of at least 24 h. To control for the potential influence of cerebral hemodynamic responses resulting from stimulant intake, participants were required to refrain from alcohol and caffeine intake for 24 h before data acquisition (Orihuela-Espina et al., 2010). During the first visit, participants were provided with an orientation of the experimental task protocol, including an introduction to each of the dumbbell exercise techniques and a brief warm-up practice for each exercise to acclimatize themselves to the performance protocol. On the second visit, the exercise protocol was reiterated to all participants, who were then allowed sufficient warm-up time before performing the experimental tasks. After the warm-up, the experimenter placed the surface electrodes over the targeted muscle belly for collecting the surface electromyography (sEMG) data, and the fNIRS probes were placed on the participants’ heads. Participants were then instructed to complete the two types of dumbbell exercise tasks per the instructions provided.

### Randomization and Blinding

The order of exercise conditions was randomized, and the experimenters were blinded to the order of conditions until the experimental visit.

### Outcome Assessment

#### Primary Outcome

**Figure 2:**
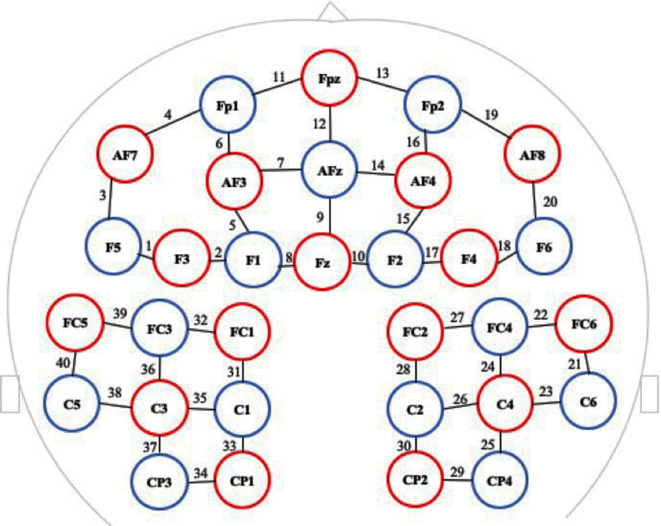
Spatial arrangement of the functional near infrared spectroscopy (fNIRS) probes in which red circles indicate the 16 optical sources, blue circles indicate the 15 detectors, and black numbers (1–40) indicate the fNIRS channels. The optical sources and detectors were positioned on the international 10-20 system.

Cerebral concentrations of HbO_2_ in the anterior frontal cortex and parietal lobe of the brain during the performance of the two exercises were the primary outcome measures. HbO_2_ was assessed during the exercise using fNIRS (NIRScout, NIRX Medical Technologies, Minneapolis, MN, USA). Participants were fitted with the fNIRS cap on their head, secured with conductive paste in the areas corresponding to their anterior frontal cortex and parietal lobe for each hemisphere (consisting of 20 channels on each side with an interoptode distance of 30 mm), and were asked to perform the exercise described in the Experimental Tasks section. Necessary adjustments, corresponding to the 10-20 system, were made to the probe for each participant (Jurcak et al., 2007). Headgear was fitted on the top of the optodes to prevent detectors from interfering with the movement. The device emitted light at two wavelengths (780 and 830 nm), with a sampling rate of 3.91 Hz. Figure 2 describes the spatial arrangement of the fNIRS probes used during the experiment.

#### Surface Electromyography

We used the Delsys data acquisition system (Trigno Wireless EMG System, Delsys, Natick, MA, USA) to record surface electromyographic signals (electrical activity) of the upper-limb muscles in response to the dumbbell exercises. The targeted muscles of the dominant arm included the anterior deltoid, posterior deltoid, biceps brachii and triceps brachii of the upper arm, and flexor carpi radialis and extensor carpi radialis longus of the forearm. Before placing the Delsys electrodes over the bellies of the targeted muscle fibers, excessive hair was shaved, and the skin was cleaned with an alcohol preparation pad and allowed time to try. After confirming signal quality, the electrodes were secured with adhesive tape and elastic straps or net bandages (Hermens et al., 2000). Raw sEMG signals were amplified, simultaneously digitized, and acquired at a sample rate of 2,000 Hz.

### Data Acquisition, Processing, and Reductions

To synchronize data acquisition, a customized synchronization box was prepared that connected the sEMG to the functional near infrared computer’s input and to the “Go” signal light.

Because HbO_2_ signals can reflect changes in the local brain blood flow better, only oxygenated concentration data were processed and analyzed (Hoshi, 2005) using the HomER2 toolbox (MGH-Martinos Center for Biomedical Imaging, Boston, MA, USA) based on MATLAB (Mathworks, Natick, MA, USA; Huppert et al., 2009). As part of the quality control, we first inspected the signal quality of the individual channels by means of the coefficient of variation, with the exclusion value set at 25% (Schneider et al., 2011). Using a movement artifact reduction algorithm, a detection of sampling points greater than 50 standard deviations from the mean was used as a rejection point for movement correction (Scholkmann et al., 2010). The data were band-pass filtered (0.01–0.1 Hz) to remove physiological noise and baseline drift (Huppert et al., 2009; Tong et al., 2012). We transformed the optical data into hemoglobin signals with mol/l based on the modified Beer–Lambert Law (Cope et al., 1988). In each channel, HbO_2_ concentration ascertained from the two dumbbell exercises was corrected by baseline (i.e., 2 s before trial onset) and then averaged for all participants (Niu et al., 2019). The mean HbO_2_ concentration for each channel was arranged in descending order with the top 25% of channels (with greatest values) defined as the channels of interest (Chen et al., 2018). The fNIRS space was converted into the standard Montreal Neurological Institute coordinate space. These channels of interest corresponded to two regions of interest (ROI) as shown in Table 1, i.e., the frontal cortex and parietal lobe.

**Table 1:** Two regions of interest (ROI).

ROI	Channel	Hemisphere	Location	Brodmann area
1	6	Left	AFp3	10-Frontopolar area
	13	Right	Fp2h	11-Orbitofrontal area
	21	Right	FCC6	43-Subcentral area
	23	Right	C6h	1-Primary somatosensory cortex
	29	Right	CP4h	2-Primary somatosensory cortex
				40-Supramarginal gyrus, part of Wernicke’s area
	30	Right	CCP2	4-Primary motor cortex
2	34	Left	CP3h	2-Primary somatosensory cortex
				40-Supramarginal gyrus, part of Wernicke’s area
	38	Left	C5h	1-Primary somatosensory cortex
	39	Left	FC5h	44-Pars opercularis, part of Broca’s area
				6-Pre-motor and supplementary motor cortex
	40	Left	FCC5	43-Subcentral area

*ROI, region of interest*.

The sEMG signals were recorded during the entire exercise at 2,000 Hz using a Delsys amplifier and data acquisition system. The measurement of sEMG signals was initially band-pass filtered at 10–400 Hz, and their means were processed using the Delsys 4.3 analysis software. For analysis purposes, we calculated the root mean square on each of the targeted muscles within 10 s.

### Statistical Analysis

Descriptive statistics were used to describe the demographic characteristics of the study participants, including age, height, weight, and frequency of weekly habitual physical activity. Differences in HbO_2_ between momentum dumbbell and conventional dumbbell exercises were assessed on the basis of the within-subject difference between the two exercise conditions with regard to the two ROIs. Accordingly, one-way repeated-measures multivariate analysis of variance (MANOVA) was conducted. In the presence of an omnibus *F*-test statistic in MANOVA, a follow-up univariate repeated-measures ANOVA was performed for each dependent variable. Statistical significance was set at *p* ≤ 0.05. A similar analytic approach was used for the six muscle outcome measures derived from the sEMG. Effect sizes are presented as partial eta-square (*η*^2^). Given the multiple dependent variables in our sEMG data, the *p*-value was adjusted for multiple testing (0.05/6 = 0.008). All data are presented as mean ± standard deviation. Statistical analyses were performed with SPSS version 25 (IBM Corp., Armonk, NY, USA).

#### Sample Size and Power

We calculated the sample size that compared the expected difference on the primary outcome of HbO_2_ in both ROIs between the two experimental (momentum dumbbell vs. conventional dumbbell exercises) conditions. On the basis of prior research (Lü et al., 2015), we determined that a sample size of 20 participants would provide 80% power (at a two-tailed *α*-level of 0.05, a correlation of 0.50) for detecting a medium effect size (Cohen’s *d* = 0.65) in HbO_2_ between the two experimental conditions in either of the ROIs. Assuming a 10% attrition rate, the study was planned for a total of 22 participants.

## Results

### Compliance and Adverse Events

A total of 23 participants were recruited and completed the experiment. There were no exercise-induced side effects observed during the study. The fNIRS data of one participant were unusable and were removed from the analysis. Thus, the data of the remaining 22 participants were analyzed and reported. Demographic characteristics of the participants are presented in Table 2.

**Table 2:** Demographic characteristics of study participants (*N* = 22).

Profile	Descriptive statistics
Age, mean (years; SD)	23.8 (2.05)
Height, mean (m; SD)	1.75 (0.06)
Weight, mean (kg; SD)	71.4 (8.80)
Habitual physical activity (days/week; SD)	4.09 (1.70)

*SD, standard deviation*.

### Primary Outcomes

**Figure 3:**
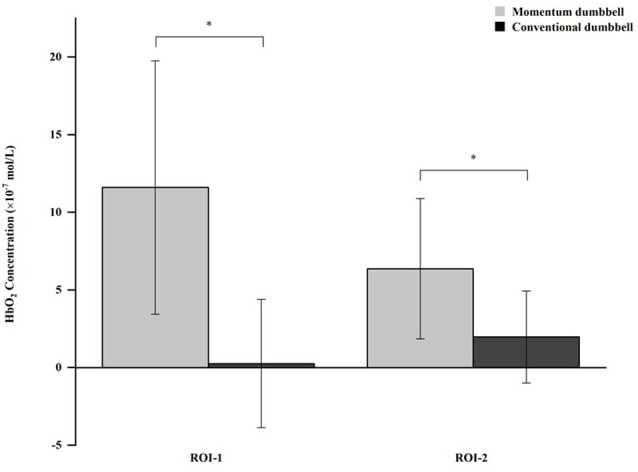
*Indicates a significant difference between the momentum dumbbell and conventional dumbbell exercises. ROI-1: Region of interest—frontal cortex. ROI-2: Region of interest—parietal lobe.

The MANOVA results showed a significant experimental condition effect on HbO_2_ across the two ROIs, *F*_(2,20)_ = 19.70, *p* < 0.001. Results from the follow-up ANOVAs showed that a significant experimental condition effect existed for both the frontal cortex (*F*_(1,21)_ = 41.34, *p* < 0.001, partial η^2^ = 0.66) and the parietal lobe (*F*_(1,21)_ = 16.96, *p* < 0.001, partial η^2^ = 0.45). An inspection of the difference in means between the two experimental conditions indicated that a statistically significant higher level of HbO_2_ concentration was observed for the momentum dumbbell exercise relative to the conventional dumbbell exercise in the frontal cortex (11.603 × 10^−7^ mol/l ± 8.159 × 10^−7^ mol/l vs. 0.257 × 10^−7^ mol/l ± 4.128 × 10^−7^ mol/l, *p* < 0.05) and in the parietal lobe (6.373 × 10^−7^ mol/l ± 4.521 × 10^−7^ mol/l vs. 1.978 × 10^−7^ mol/l ± 2.962 × 10^−7^ mol/l, *p* < 0.05). Figure 3 displays the differences in HbO_2_ concentrations in the frontal cortex and parietal lobe between the momentum and conventional dumbbell exercises.

### Secondary Outcomes

**Figure 4:**
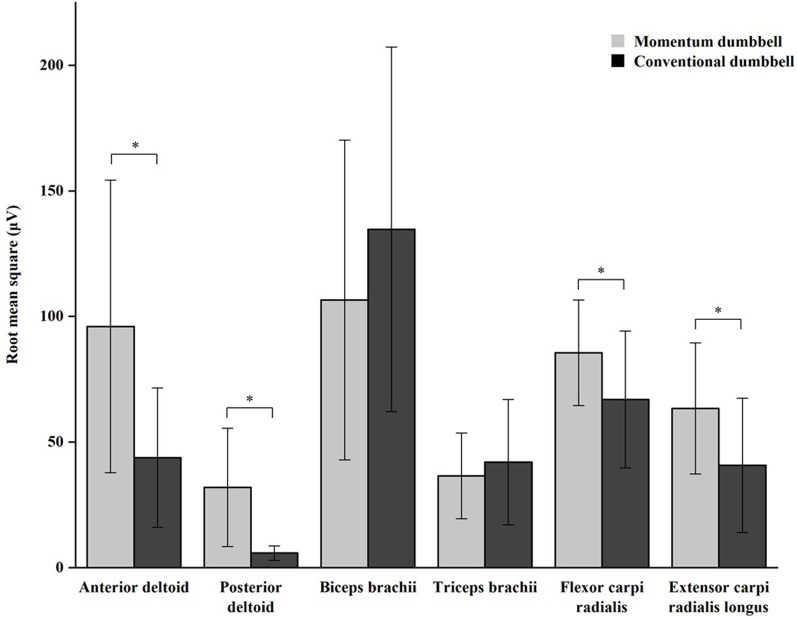
*Indicates a significant difference between the momentum dumbbell and conventional dumbbell exercises.

The MANOVA results indicated a significant experimental condition effect on the six muscles studied, *F*_(6,16)_ = 20.65, *p* < 0.001. Of the six ANOVAs conducted, four were statistically significant (*p* ≤ 0.003). Compared to the conventional dumbbell exercise, momentum dumbbell exercise was shown to involve a higher level of electrical activity in the muscles of the anterior and posterior deltoids of the upper arm and the flexor carpi radialis and extensor carpi radialis longus of the forearm (*p* < 0.05). There was, however, no significant difference in the biceps brachii (*p* = 0.06) and triceps brachii (*p* = 0.37). Estimates on sEMG are presented in Figure 4.

## Discussion

In this study, we compared the acute effects of two resistance-based dumbbell training exercises on cerebral cortical activation among healthy young male adults. Through quantification of fNIRS, we found that, although both the momentum and conventional dumbbell exercises elicited oxygenated hemodynamic responses in the frontal cortex and parietal lobe, the momentum dumbbell exercise produced a higher level of acute cerebral response than the conventional exercise. In addition, relative to the conventional exercise, performing the momentum exercise led to a higher level of muscle activation in the anterior and posterior deltoids of the upper arm and flexor carpi radialis and extensor carpi radialis longus of the forearm.

The results regarding the hemodynamic responses observed during both dumbbell exercises in this study are consistent with findings showing hemodynamic responses during walking (Miyai et al., 2001), running (Suzuki et al., 2004), cycling (Lin et al., 2012, 2013; Tempest and Reiss, 2019), and video game-based dynamic balancing exercise (Karim et al., 2012) among healthy adults and patients with stroke. However, this was the first study that compared two active but different resistance training regimens, with the results showing a higher level of hemodynamic responses elicited from the momentum-based dumbbell exercise. Using the fNIRS neuroimaging tool, the study extended the findings of a previous momentum dumbbell intervention study (Lü et al., 2015) by showing that the momentum dumbbell exercise was also associated with hemodynamic alterations as evidenced in both the frontal cortex and parietal lobe, areas in the brain that are considered important for cognitive functioning and the executive control of arm movement (Rossi et al., 2009).

The exact mechanisms by which the momentum dumbbell exercise elicits the hemodynamic response are unclear. One plausible explanation is the challenging movement characteristics of this novel exercise modality. The exercise requires that an individual perform with a high level of coordination between upper-extremity strength and core control, as well as attention necessary to maintain the spinning action of the dumbbell (i.e., spinning of the pendulum inside the dumbbell). This dual-tasking feature may have been responsible for the increased level of HbO_2_ observed in the frontal cortex and parietal lobe, findings that align with the hemodynamic responses observed in aerobic-based exercise with dual tasking (Mirelman et al., 2014). In contrast, conventional dumbbell exercise primarily involves a single (elbow) joint arm extension/flexion mechanical movement performed in a single (sagittal) plane. Naturally, we cannot exclude the possibility that performance of the momentum, relative to the conventional, dumbbell exercise may require higher effort by the participants, inducing higher work load and thus increasing activation. Nevertheless, the current findings provide impetus for future inquiries regarding the mechanisms by which resistance-based exercises induce cerebrovascular changes.

In addition to the increase in the level of HbO_2_ in response to the dynamic dumbbell exercises, our EMG analyses showed high levels of muscle activity involving the shoulder and forearm during dumbbell performance. The differences in muscle activation patterns between the two exercise formats may have been due to the high speed and frequency of movements occurring during the momentum dumbbell exercise, resulting in high muscle recruitment, whereas for the conventional dumbbell exercise, the elbow joint of the performing arm was relatively fixed while engaging in bicep curls (flexion-extension), with limited active engagement of the proximal (shoulder) and distal (wrist) joints. Collectively, these EMG data provide additional support for the hemodynamic responses observed because they indicate concomitant activities occurring both at the cortical level and in the working muscles involved in the performance of the experimental dumbbell task, with higher levels of muscle activity observed in the momentum dumbbell exercise. Such a co-occurring event, however, does not necessarily imply that one is the cause of the other (i.e., that cortical activation causes the muscle activities in question). We observed no difference between the two exercise methods in the biceps and triceps brachii of the upper arm. While the dumbbell exercise movement predominantly involves bicep curls, our results suggest that there is an equal amount of involvement or firing in the biceps brachii while performing the exercises with either type of dumbbell.

This study adds new knowledge regarding a novel momentum-based dumbbell exercise, compared to a conventional dumbbell exercise, in inducing change in cerebral cortical activation, which will allow us to advance exercise-cognition research (Erickson et al., 2007; Voss et al., 2010). With the use of neuroimaging techniques, such as fNIRS, the findings of this study provide a premise to explore the neural correlates of exercise, establish a bridge between brain activity and cognitive and motor functions, and elucidate the neural systems associated with exercise and cognition. From an exercise-enhancing perceptive, future research may focus on examining the potential value of the momentum dumbbell exercise on altering brain neural activity using complex movement configurations and different exercise intensities to enhance brain function (Lü et al., 2015).

### Strengths and Limitations

A notable strength of our study is the use of fNIRS for the real-time examination of cerebral cortical activation induced by the momentum-based dumbbell exercise. The study, however, has some limitations. First, the participants in the study were healthy male college students, rendering generalization of the results to women and other populations difficult; this is especially relevant for older adults whose brain health may benefit most from this type of exercise (Lü et al., 2015). Second, the current study design only involved experimental conditions (dumbbell exercises) without evaluation at two different time points (e.g., pre- and post-activity evaluation), thus limiting the ability to make an inference regarding changes in hemodynamic responses from baseline. Third, although we ensured that the performance time and workload were constant across the two conditions (10, 10-s sets of exercise), we did not measure the intensity of either exercise during the experiment. It is likely that given the novelty of and performance difficulties in maintaining the momentum dumbbell spinning, more effort may have been exerted by the participants while performing the momentum dumbbell task. This may have resulted in higher intensity for the momentum, relative to the conventional, dumbbell exercise, leading to increased activation. Finally, given the lack of measures of cognitive function in the current study, it was impossible to evaluate the associations between exercise-induced brain activation and cognitive performance.

### Conclusions

A momentum, compared to a conventional, dumbbell exercise was shown to be effective in eliciting hemodynamic responses and muscle activation of the upper limbs among healthy young male adults. Future investigators analyzing this form of exercise should focus on exploring the therapeutic value of this new exercise modality in clinical populations (i.e., patients with cognitive and/or movement impairment), examining the long-term effects, and investigating the link between hemodynamic responses and cognitive function.

## Data Availability Statement

All datasets generated for this study are included in the article.

## Ethics Statement

The studies involving human participants were reviewed and approved by Shanghai University of Sport. The patients/participants provided their written informed consent to participate in this study. Written informed consent was obtained from the individual(s) for the publication of any potentially identifiable images or data included in this article.

## Author Contributions

YW, LH, and YL: study concept and design. YW, LS, WW, and YJ: acquisition of data. YW, JL, and JR: analysis and interpretation. YW: drafting of manuscript. LH and YL: critical revision of manuscript and study supervision. LH: obtain funding. All authors contributed to the article and approved the submitted version.

## Conflict of Interest

The authors declare that the research was conducted in the absence of any commercial or financial relationships that could be construed as a potential conflict of interest.

## Abbreviations

fNIRS: functional near infrared spectroscopy
HbO_2_: oxygenated hemoglobin
MANOVA: multivariate analysis of variance
sEMG: surface electromyography.
